# Use of qPCR-Based Cercariometry to Assess Swimmer’s Itch in Recreational Lakes

**DOI:** 10.1007/s10393-018-1362-1

**Published:** 2018-08-17

**Authors:** Sydney P. Rudko, Ronald L. Reimink, Kelsey Froelich, Michelle A. Gordy, Curtis L. Blankespoor, Patrick C. Hanington

**Affiliations:** 1 School of Public Health, University of Alberta, Room 3-57, South Academic Building, Edmonton, AB T6G 2G7 Canada; 2 Office of Campus Ministries, Hope College, Holland, MI USA; 3 Saint Joseph High School, Saint Joseph, MI USA; 4 Jackson College, Jackson, MI USA; 5 University of Michigan Biological Station, Pellston, MI USA

**Keywords:** Swimmer’s itch, qPCR, Recreational water, Exposure, Cercariometry, Avian schistosomes, eDNA

## Abstract

Swimmer’s itch (cercarial dermatitis) is a nuisance encountered by bathers and recreational water users worldwide. The condition is caused by the penetration of larval digenean trematodes (cercariae) of the family Schistosomatidae, into the skin, following their release into freshwater from pulmonate snails that serve as the intermediate hosts for these parasites. This study utilizes qPCR-based cercariometry to monitor and quantify cercariae from water samples collected at 5 lakes in northern Michigan. The resolution provided by qPCR facilitated assessment of the environmental and biological drivers of swimmer’s itch-causing cercariae concentrations, allowing us to demonstrate that cercarial abundance is greatest at the top of the water column, in locations with prevailing on- and alongshore winds.

**Electronic supplementary material:**

The online version of this article (10.1007/s10393-018-1362-1) contains supplementary material, which is available to authorized users.

## Introduction

Parasites of the family Schistosomatidae utilize avian or mammalian definitive hosts and snail intermediate hosts to complete their life cycles. Adult worms reside in the avian or mammalian hosts and produce embryonated eggs that typically exit the definitive host via feces or urine into the aquatic environment, where they hatch into a free-living larval stage (miracidium). Miracidia penetrate the snail host and develop into sporocysts. Sporocysts produce cercariae asexually, which are then shed from infected snails (Cort 1928; Blair and Ottesen 1979; Blair and Islam 1983). Cercariae then seek out their definitive host to complete their life cycle. The number of cercariae released each day can vary significantly between hundreds to tens of thousands of larval parasites and can be influenced by factors such as temperature, duration of infection, and immunogenetic determinants of snail-schistosome compatibility (Sluiters et al. 1980; McCarthy 1999; Zbikowska 2004; Coady et al. 2006; Gordy et al. 2015; Soldánová et al. 2016; Pila et al. 2016). While the cercariae of some species of schistosome (those of the genus *Schistosoma*) are known to infect humans, species of the genera *Trichobilharzia, Gigantobilharzia, Dendritobilharzia, Anserobilharzia,* and *Schistosomatium* cannot, but are able to penetrate human skin and lead to a condition known as swimmer’s itch (Cort 1928; Batten 1956; Kolářová et al. 1999).

Swimmer’s itch, or cercarial dermatitis, is caused by epidermal penetration of humans by larval cercariae in surface waters (Brant and Loker 2009). The resultant immune response leaves itchy papules that can last for weeks (Baird and Wear 1987; Verbrugge et al. 2004; Soldánová et al. 2013). Swimmer’s itch is a prominent problem in recreational lakes in northern Michigan, where the predominant species responsible for swimmer’s itch is *Trichobilharzia stagnicolae*, hosted by the common merganser (*Mergus merganser*) and the snail *Stagnicola emarginata* (Blankespoor and Reimink 1991; Keas and Blankespoor 1997).

Monitoring efforts for swimmer’s itch typically rely on assessments of infection prevalence of relevant parasites in snails. This method requires collecting a large number of snails from multiple sites around a lake and allowing the snails to shed parasites. Shed cercariae are then morphologically identified using a stereomicroscope. While this method assesses the success of a specific parasite species in infecting a snail species such as *S. emarginata,* it has a number of downsides. Notably, avian schistosomes typically show a low prevalence in snail intermediate hosts (Crews and Esch 1986; Brown et al. 1988; Gordy et al. 2016), making detection difficult, and collecting and screening snails is time-consuming. Furthermore, as cercariae are essential both for transmission of the parasite, and cause swimmer’s itch, quantifying their abundance in the water is key to understanding both the ecological and public health aspects of this parasite. A quantitative polymerase chain reaction assay for the detection of avian schistosomes, or swimmer’s itch-causing cercariae, in recreational water was validated and published in 2015 (Narayanan et al. 2015). This assay can detect species of the family Schistosomatidae, including *Trichobilharzia* spp., *Schistosomatium douthitti*, *Allobilharzia* spp., *Gigantobilharzia,* spp., and *Dendritobilharzia* spp. qPCR cercariometry offers a notable advantage to traditional snail infection prevalence methodologies when the end goal is assessment of swimmer’s itch risk. Cercariae concentration and distribution in the water body may be influenced by environmental factors such as wind and water movement, or predation, inactivation due to UV light, or death (Haas 1994; McCarthy 1999). As qPCR cercariometry enables the quantification of cercariae in the water and at recreation sites, it measures the potential exposure of bathers to cercariae. Some questions still remain regarding the accuracy and reproducibility of using qPCR to measure trematodes (or for that matter, eukaryotic organisms) in water. Additionally, approaches to quantify to an organismal level remain in their infancy for eukaryotic targets.

In this study, we have expanded upon qPCR cercariometry as a method to quantify cercariae abundance and use it to monitor for avian schistosome cercariae in lakes in northern Michigan. This paper defines appropriate sample collection methodology, and documents methods for estimating DNA losses due to DNA extraction, which enables a robust estimation of the number of cercariae present in samples. We have assessed the accuracy and reproducibility of the method in controlled laboratory experiments, and in the field. Furthermore, we have applied this method to monitor for swimmer’s itch-causing cercariae at a variety of lakes in northern Michigan, and used these data to assess the effects of wind direction, time of day, and seasonality on cercariae abundance.

## Methods

### Environmental Sampling

#### Water Sampling

Twenty-five-liter water samples were collected at each location and passed through a 20 cm × 80 cm × 20 micron zooplankton net (Aquatic Research Instruments). Samples were collected liter by liter across the entire swath of the beach, in approximately waist deep water, with the goal of maximizing the likelihood of capturing free-swimming cercariae. Debris from inside the net were washed down using well water followed by a 95% ethanol wash and collection in 50-mL conical tubes. This 50-mL sample was then passed through a 0.45-µM polycarbonate filter (Pall), the filter paper removed from the bed, and stored in 1 mL of 95% ethanol for transport to the University of Alberta (Canada) for DNA extraction and qPCR analysis.

#### Environmental Variable Measurements

Wind direction, and effect (i.e., onshore, offshore, alongshore), as well as with time of day were also recorded at each sampling site.

#### Snail, Miracidia, and Cercariae Collection

Cercariae, miracidia, and snails were collected from Higgins Lake, Lake Leelanau, and Crystal Lake in 2016. Cercariae shed from snails were pipetted into collection tubes and preserved with 95% ethanol. Tissue from each representative snail species was also preserved in 95% ethanol. Miracidia were collected from fresh bird feces deposited on the beach by diluting fecal samples in well water, hatching the miracidia under fluorescent lights, and pipetting into 95% ethanol. The species and approximate age of the bird from which the feces were deposited were recorded at the time of collection. Ethanol preserved samples were stored at ~ − 10 °C and then cold-shipped to the University of Alberta for further processing.

#### Method Robustness

Three consecutive samples (25 L each) at 10 sampling locations on Crystal Lake were collected and evaluated to assess the precision of the entire method in the field. Additionally, three samples containing an unknown number of cercariae were sent from researchers in Michigan to the University of Alberta where qPCR was performed in a single blind trial to assess the accuracy of the qPCR assay in predicting the true number of cercariae in a sample.

#### Time of Day Sampling

Eight locations were selected on Big and Little Glen Lakes for sampling on July 25, 2017. Two teams collected water samples at each of 4 locations at approximately 8:00, 8:15, 8:30, and 8:45 a.m. The 4-site collection sequence was repeated beginning at 12:00 p.m., 4:00 p.m., and 8:00 p.m.

#### Depth of Water Sampling

A stacked water column trap was designed and built to capture stacked subsamples of water. The water column trap can be used at depths up to 1.5 m deep. The trap consists of 3, 4-inch PVC ball valves connected in series by 2, 4 × 6 inch threaded nipples (Menards #MA0013H, #CC08350). Each section corresponded to a depth of 50 cm. The column trap was lowered into the water with valves open. Each valve was then manually closed, trapping 550 mL of lake water in each connector nipple. The subsamples were then emptied into separate buckets by opening the valves, one by one beginning at the bottom. A total of 30 full columns were collected. The buckets were then emptied into a 20-μm mesh plankton tow, the net washed with 95% ethanol, and the sample concentrated to ~ 50 mL. The depth of the water where sampling occurred was approximately 1.2 m. Therefore, the topmost section of the trap was not completely covered by the water, and the topmost section of the trap only collected the top 30 cm of the water column (Supplementary Figure 1).

### Molecular Methodologies

#### DNA Sequencing

DNA extraction, PCR amplification of the Cytochrome *c* oxidase subunit 1 gene (*cox1*), Sanger sequencing, and alignments were completed as described in Gordy et al. (2016). The *cox1* mitochondrial DNA sequences generated in this study are available on NCBI GenBank from accession numbers MG964019 to MG964043.

#### Extraction of Schistosome DNA from Water Samples

In 2016, ethanol was evaporated from filters using a vacuum centrifuge. Subsequently, 200 µL of lysis buffer AL (Qiagen) and 20 µL of proteinase K were then added to the tube containing the filter, along with 1-mm silica carbide beads, and bead beat for 10 min on high using a vortex before DNA extraction (as described by Webster (2009), using the DNeasy Extraction Kit [Qiagen]). In 2017, DNA extraction was accomplished in Michigan using the Biomeme Environmental DNA extraction kit (Biomeme, Philadelphia, PA, USA) according to the manufacturers instructions, with one deviation—samples were eluted into 100 µL of elution buffer. This change was made to reduce costs associated with shipping samples as the Biomeme extraction can be accomplished lakeside in Michigan.

#### qPCR Detection of Schistosome *18S* rRNA

The *18S* rRNA qPCR assay was run according to the method described in Narayanan et al. (2015). The sequences of the forward, probe, and reverse primer are, respectively, AGCCTTTCAGCCGTATCTGT, TCGGGAGCGGACGGCATCTTTA, AGGCCTGCCTTGAGCACT. Unlike the original method, samples were quantified to copies of plasmid DNA using a standard curve that consisted of 50,000, 5000, 500, 50, and 0.5 copies of cloned puc57 plasmid DNA (Genscript, New Jersey, USA) containing the avian schistosome *18S* rRNA gene. The LOD_95_ of this technique is 3.4 copies per reaction (upper limit 7.9, lower limit 1.5) (Wilrich and Wilrich 2009). Samples and standards were performed in triplicate, with inhibition controls run for each sample in duplicate. Inhibition controls were performed as described in Rudko et al. (2017), where a plasmid inhibition control was spiked into water samples and subsequently detected by qPCR. Inhibition was defined as a 3-cycle threshold shift (USEPA 2012). Thermocycling (qPCR) was performed in a post-amplification room using the ABI 7500 Fast Real Time qPCR system.

#### Quantitation of Plasmid Copies Per Cercaria

**Figure 1:**
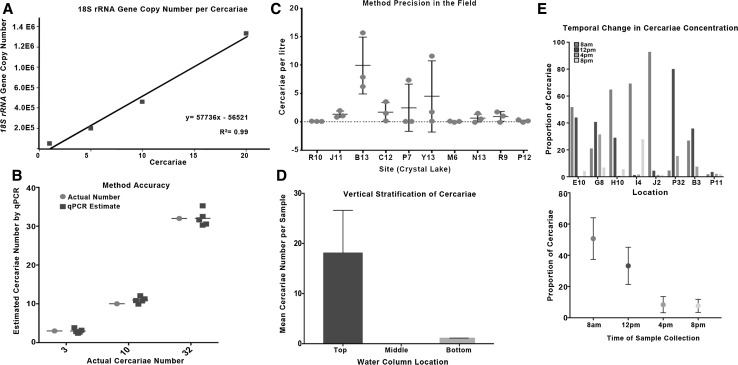
qPCR Method Validation. **A** Cercariae standard curve used to derive conversion equation for the conversion of qPCR copy number to number of cercariae. **B** Accuracy of the qPCR method. Actual number of cercariae, hand counted via microscopy, and the qPCR estimate of cercariae based on five replicate qPCR reactions. **C** Precision of the method in the field. Replicate 25-L water samples were analyzed at different sites on Crystal Lake in 2016. Error bars represent standard deviation around the mean. **D** Vertical stratification of cercariae concentrations. Top = 0–30 cm from surface, Middle = 50–100 cm, Bottom = 100–150 cm. e. Daily changes in cercariae concentration. Top: Changes in proportion of cercariae at each site from 8 a.m. to 8 p.m. Bottom: Average proportion of cercariae of all sites at each time point.

Hand counted stocks (stereo microscope [Zeiss]) of *T. stagnicolae* cercariae (1, 5, 10, and 20 cercaria(e)) were counted, and DNA extracted, according to Webster (2009). The average *18S* rRNA copy number was determined using qPCR. Results were used to generate a standard curve to convert DNA copies per sample to cercariae per sample. Conversion equation: *x *= [(*y* + 56521)/57736] was derived and used to calculate the number of cercariae per sample. *X* is the number of cercariae, and *y* is the copy number per sample (Fig. 1A).

#### Extraction Efficiency

Extraction efficiency was determined by spiking 5 *T. stagnicolae* cercariae onto a clean 0.45-µM filter membrane and preserving the samples in 100% ethanol. Samples were analyzed in the same manner as described above for the water samples. Conversion equation was applied to determine the number of cercariae recovered through the method under ideal circumstances.

### Statistics

The change in cercariae concentrations relative to the time of day, and wind direction relative to the shoreline, was assessed using a generalized linear model with a log link function, based on the negative binomial distribution. The outcome variable was cercariae concentration. Explanatory variables were lake, week (standardized to account for different days during the same week across years), time of day (morning or afternoon), and wind direction (onshore, offshore, and alongshore). In the initial model, the interaction effect of time of day and wind direction was also tested, but this interaction was found nonsignificant and was removed to improve model fit. Additionally, site on the lake, year, and lake were modeled as random effect variables in the original model with independent correlation matrices, but were found to be insignificant and were removed from the final model. All statistical analyses were performed using SPSS (version 24, IBM, Armonk, North Castle, New York, USA). T tests and graphs were executed using PRISM (ver. 7.0, GraphPad).

## Results

### Snail and Trematode Species Identifications

Mitochondrial *cox1* DNA sequences confirmed *T. stagnicolae* (99.4–100% matches to GenBank vouchers) to be the common schistosome emerging from *S. emarginata* among Higgins Lake, Crystal Lake, and Lake Leelanau. Miracidia from *M. merganser* (common merganser duck), *Anas platyrhynchos* (mallard duck), and *Branta canadensis* (Canada goose) were collected. As suspected, *Trichobilharzia* sp. miracidia were found in the feces of mergansers and mallards. A miracidia, which most closely matched to a member of the Schistosomatidae family, was found in Canadian geese. The avian trematode *Dendritobilharzia* sp. miracidia were also found in the feces of mallard ducks (Table 1) (Brant et al. 2011).

**Table 1:** Snail, Miracidia, and Cercariae Species Identified Based on Cox-1 Sequencing from Higgins Lake, Crystal Lake, and Lake Leelanau in 2016.

Identification	Pairwise similarity (%)	Accession match
Crystal Lake		
Cercariae		
*Diplostomum* sp*. 1*	100.0	KR271221.1
*Plagiorchis* sp.	99.3	FJ477214.1
*Apatemon* sp. 1	90.6	HM064617.1
*Trichobilharzia Stagnicolae*	100.0	FJ174489.1
Snail		
*Physella ancillaria*	99.56	KM612195.1
Unclassified Planorbidae	100.00	KM612069.1
Miracidia		
*Trichobilharzia stagnicolae* From hatch-year common merganser brood	99.78	KT831352.1
*Trichobilharzia stagnicolae* From hatch-year common merganser brood	100	KT831352.1
*Trichobilharzia stagnicolae* From hatch-year common merganser	99.38	FJ174490.1
Higgins Lake		
Cercariae		
*Diplostomum sp. 4*	99.2	KR271383.1
*Apatemon sp. 1*	90.6	HM064617.1
*Plagiorchis sp.*	99.5	FJ477214.1
*Stagnicola elodes*	97.8	KM612224.1
Snail		
*Physella ancillaria*	99.84	KM612168.1
*Elimia livescens*	100	EF586916.1
*Campeloma decisum*	99.6	KU905792.1
*Stagnicola elodes*	97.8	KM612224.1
*Marstonia lustrica*	99.7	AF520945.1
Miracidia		
*Trichobilharzia stagnicolae* From hatch-year common merganser	99.8	KT831352.1
Lake Leelanau		
Cercariae		
*Trichobilharzia stagnicolae*	99.8	FJ174490.1
Snails		
*Stagnicola elodes*	97.7	KM612224.1
Unclassified Planorbidae	99.8	KM612069.1
*Stagnicola elodes*	99.7	HQ969867.1
*Physella gyrina*	98.5	KT708102.1
*Pleurocera catenaria*	83	EU414649.1
*Unidentified Planorbidae*—*Biomphalaria choanomphala/Biomphalaria tenagophila*	85	HM768933.1/EF433576.1
Miracidia		
*Trichobilharzia stagnicolae* From hatch-year common merganser	99.80	FJ174492.1
*Dendritobilharzia sp.* From hatch-year mallard	99.0	KX302892.1
*Schistosomatidae gen. spp* From Canadian goose.	84.0	FJ174486.1

### qPCR Method Validation

#### qPCR Validation Against Trematode Library

The qPCR assay was tested against a library of 25 North American trematodes (Gordy et al. 2016). Amplification was only observed in samples containing *Trichobilharzia* sp. (Table 2).

**Table 2:** The qPCR Assay is Specific to Swimmer’s Itch-Causing Cercariae.

Species	qPCR Result
Trichobilharzia stagnicolae*	+
Trichobilharzia szidati*	+
*Cotylurus sp.*	−
*Diplostomum baeri*	−
*Diplostomum huronense*	−
*Diplostomum indistinctum*	−
*Diplostomum sp. 1*	−
*Diplostomum sp. 2*	−
*Diplostomum sp. 3*	−
*Diplostomum sp. 8*	−
*Drepanocephalus auritus*	−
*Echinostoma caproni*	−
*Echinostoma trivolvis*	−
*Neodiplostomum americanum*	−
*Notocotylidae sp.*	−
*Haematoloechus sp.*	−
*Icthyocotylurus sp. 3*	−
*Ornithodiplostomum sp. 8*	−
*Plagiorchis sp.*	−
*Pseudopsilostoma varium*	−
*Strigeidae gen.*	−
*Telorchis sp.*	−
*Apharyngostrigea pipientis*	−
*Bolbophorus sp.*	−
*Tylodelphys scheuringi*	−

The qPCR assay was tested against a library of purified trematode DNA from across North America. Asterisks indicate swimmer’s itch-causing species. (+) indicates the target product amplified, while (−) indicates that no target was amplified.

*Statistically significant (*P* < 0.05)

#### Extraction Efficiency

DNA losses occur during any DNA extraction due to inefficient lysis or DNA absorbance. DNA extraction efficiency using the DNeasy kit ranged from 0.1 to 7%, and averaged 4.2%. DNA extraction efficiency using the Biomeme eDNA kit employed during the 2017 collection year averaged 4.1%, and ranged from 0.4 to 10%. These values are not significantly different (*p* [*two tailed*] = 0.6, *df* = 19). Copy numbers were adjusted to reflect a recovery rate of 4.2% (Table 3).

**Table 3:** Extraction Efficiencies of the Qiagen DNeasy DNA Extraction Kit, and the Biomeme eDNA Field Extraction Kit.

Trial	Loss (%)	Recovery (%)
Qiagen DNeasy		
1	99.99	0.01
2	99.2	0.8
3	99.9	0.1
4	98.9	1.1
5	98.7	1.3
6	92.2	7.8
7	92.0	8.0
8	99.7	0.3
9	99.7	0.3
10	98.0	2.0
11	98.0	2.0
12	92.8	7.2
13	94.3	5.7
14	92.9	7.1
15	92.9	7.1
Biomeme eDNA		
1	98.1	1.9
2	96.1	3.9
3	98.6	1.4
4	78.8	21
5	96.6	3.4
6	99.0	1.0
7	98.7	1.3
8	99.6	0.40

#### Accuracy and Precision of the qPCR Method

**Figure 2:**
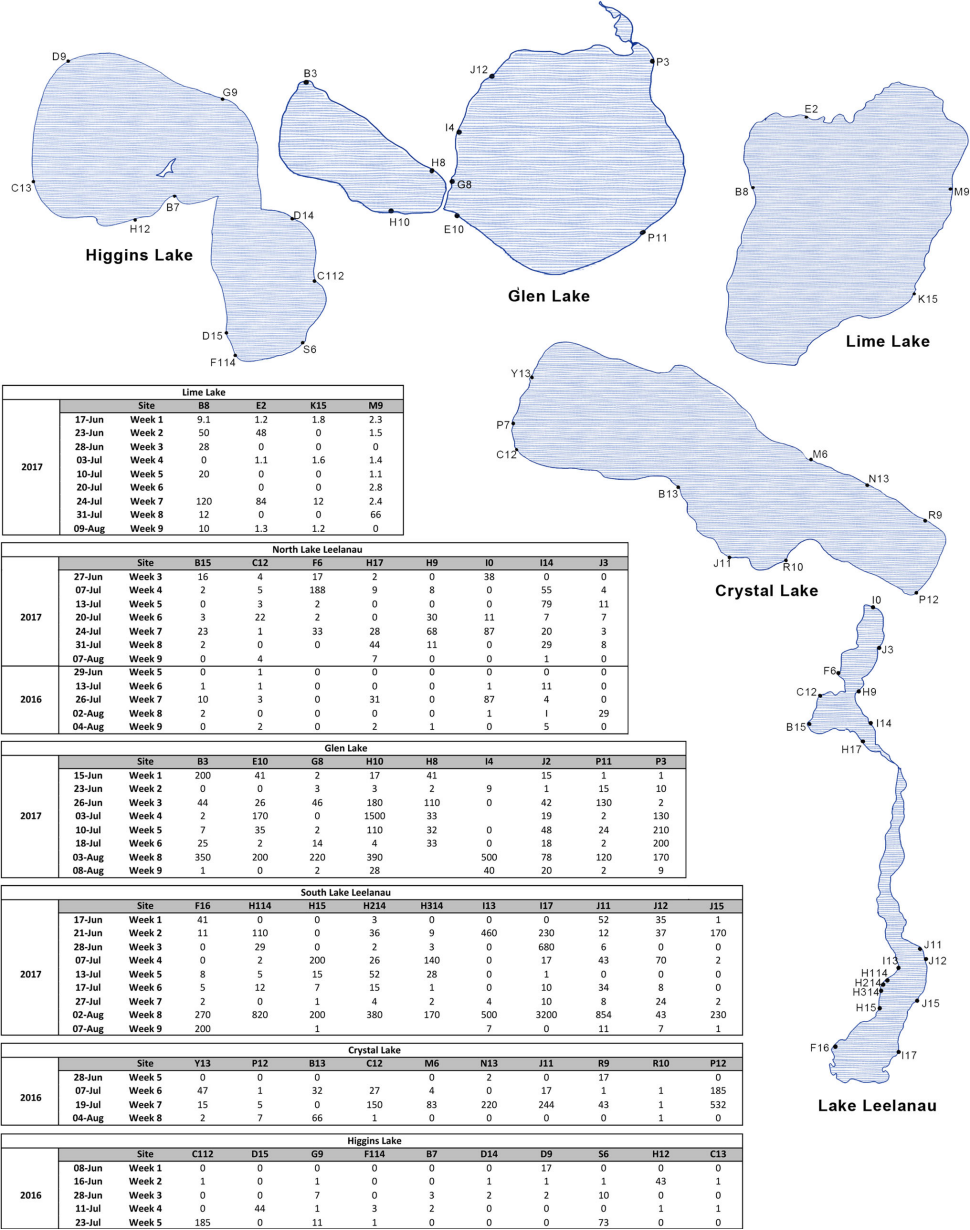
Monitoring results. Lakes in northern Michigan were monitored weekly throughout 2016 and 2017. Results are in cercariae/25 L.

Sampling variability was assessed by three samples taken consecutively of 25-L water samples at ten beaches on the same day on August 1, 2016, from Crystal Lake (Fig. 2). The sites with the highest dispersion between consecutive samples (Sites B13, P7, Y13) differed by 10, 8, and 12 cercariae each, respectively (Fig. 1C). Additionally, a single blinded experiment was performed wherein known cercariae stocks were shipped from Michigan, USA, to the laboratory in Alberta, Canada to be analyzed. The DNeasy extraction, qPCR, coupled with analysis using conversion equation correctly predicted the number of cercariae in each stock all three times the experiment was performed (Fig. 1B). The copy number in a single *T. stagnicolae* cercariae averages 55,851 copies (*σ*_*x*_ = 65.85).

### Environmental and Seasonal Drivers of Cercariae Abundance

#### Avian Schistosome Concentrations Vary in the Water Column

The vertical distribution of cercariae in the water column was assessed and demonstrates that the majority of swimmer’s itch-causing cercariae position themselves in the top 30 cm of the water column (Fig. 1D).

#### Time of Day Sampling

Eight (25 L) samples were collected at 4 time points throughout the day on July 25, 2017, to determine if the time of sample collection influences cercariae counts. On average, the highest numbers of cercariae were detected in the water at 8 a.m., and average numbers of cercariae at the same sampling point declined throughout the day (Fig. 1E).

#### Monitoring Results

Monitoring projects were conducted in 2016 and 2017. In 2016, Higgins Lake (44.495790, − 84.741729), Crystal Lake (44.661876, − 86.170675), and Lake Leelanau (44.981424, − 85.711727) were monitored, and in 2017, Lake Leelanau, Lime Lake (44.896239, − 85.841167), and Glen Lake (44.864639, − 85.962053) were monitored. Sampling sites were selected based on local knowledge—places where property owners commonly reported swimmer’s itch were sampled. South Lake Leelanau, Glen Lake, and Crystal Lake saw the highest numbers of cercariae detected in the water throughout their respective sampling periods, while Higgins, Lime Lake, and North Lake Leelanau have lower numbers of cercariae. However, it is important to note the high degree of variability in cercariae concentrations among all lakes; even lakes with lower concentrations of cercariae occasionally see extreme peaks in cercariae concentration, likely due to environmental factors such as wind or beach hydrology, which may concentrate cercariae in certain locations (Fig. 2).

#### Wind Direction Relative to the Shoreline Predicts Cercariae Concentrations

The effect of the factors, such as time of day, wind direction relative to the shore, and week (standardized between years, for exact dates see Fig. 2), was tested on cercariae concentrations. These effects were tested using a generalized linear model (Table 4). Results indicate that onshore and alongshore winds are predictive of higher concentrations of cercariae. The data also show that cercariae concentration changes temporally over the summer months, with weeks 4 and 8 of the sampling period predicting higher numbers of cercariae.

**Table 4:** Generalized Linear Model Analysis of Environmental Factors Influencing Cercariae Concentrations.

Main effects	*P* value
Wind direction	< 0.0001*
Lake	< 0.0001*
Week	< 0.0001*
Time of day (am/pm)	0.59

Parameter	*β*	Confidence interval	*P* value
Parameter estimates			
Wind			
Onshore wind	0.89	0.23–1.5	0.008*
Alongshore wind	1.2	0.57–1.8	< 0.0001*
Offshore wind	0.48	− 0.24 to 1.2	0.19
Lake			
North Leelanau	− 1.213	− 1.7 to − 0.7	< 0.0001*
Lime Lake	− 1.825	− 2.3 to − 1.3	< 0.0001*
Higgins	− 1.406	− 0.77 to − 0.72	< 0.0001*
Glen Lake	− 0.077	− 0.47 to 0.14	0.70
South Leelanau	− 0.045	− 0.42 to 0.50	0.85
Crystal	0.045	− 0.42 to 0.50	0.85
Week			
Week 1	− 0.74	− 1.5 to 0.05	0.07
Week 2	− 0.58	− 1.2 to 0.70	0.08
Week 3	− 0.13	− 0.75 to 0.50	0.70
Week 4	0.63	0.03 to 1.23	0.04*
Week 5	− 0.12	− 0.8 to 0.50	0.72
Week 6	− 0.42	− 1.2 to 0.30	0.25
Week 7	0.38	− 0.43 to 1.2	0.36
Week 8	2.53	1.9 to 3.2	< 0.0001*
Week 9	0.738	− 0.58 to 0.9	0.07
Time of day			
Morning (8 a.m.–12 p.m.)	0.08	− 0.20 to 0.36	0.60
Afternoon (12 p.m.–8 a.m.)	0.08	− 0.20 to 0.36	0.60

*Statistically significant (*P* < 0.05)

## Discussion

The goals of this study were twofold: to aid in the validation of qPCR detection of avian schistosomes, and to utilize the assay in monitoring projects to assess the effects of environmental variables on the distribution of cercariae in the water.

### Method Validation

The common merganser has long been assumed to contribute the majority of avian schistosomes to the lakes in northern Michigan (Blankespoor and Reimink 1991), and our survey confirms this observation. We also report *Dendritobilharzia* spp. miracidia, another schistosome species capable of causing swimmer’s itch in the feces of mallard ducks (Table 1). The qPCR assay in its initial validation by Narayanan et al. (2015) was designed to detect all species of the family Schistosomatidae, which includes *Trichobilharzia* and *Dendritobilharzia* spp.

qPCR has become a well-vetted and well-established method for recreational water monitoring for enteric bacteria (USEPA 2012); however, it is still an emerging methodology in parasitology. Molecular methods for the detection of the environmental stages of parasites have become more common in recent years and can be applied to fill crucial knowledge gaps related to the environmental transmission stages. As a review by Bass et al. (2015) points out, environmental sampling for parasites coupled with molecular methods can reduce sampling bias incurred by specimen-based sampling. Additionally, molecular parasitological methods have been demonstrated to be more cost-effective, less labor intensive than specimen-based detection (Huver et al. 2015). However, there are a number of limitations, notably sample inhibition, DNA losses, and difficulties in quantifying to the organismal level, and the inability to distinguish between live and dead organisms. Eukaryotic targets in qPCR can be more challenging to validate because they are multi-cellular, and the *18S rRNA* gene target is multi-copy within the genome (Weiss et al. 2017; Bik et al. 2013). Ribosomal gene copy numbers are known to vary by orders of magnitude in bacteria and fungi, and apparent copy numbers quantified by qPCR may also be influenced by downstream methods such as losses due to DNA extraction (Cankar et al. 2006; Adamska et al. 2010; Green and Field 2012). Given this, quantitation to an organismal level for a eukaryotic target must include a conversion between gene copy number and organismal number, which is predicated on an assessment of DNA losses due to extraction. The majority of the Michigan lakes studies were relatively pristine, oligotrophic lakes. Silt and sandy debris in the water samples occasionally presented a challenge, but could be overcome by decanting off the liquid portion of a sample, leaving the debris behind before extraction. Procedures to the DNA extraction protocols may need to be modified if a similar study was replicated in a mesotrophic or eutrophic environment, or in the presence of cyanobacteria or algal blooms.

The average *18S* rRNA gene copy number in a single cercaria is 55,851 copies; however, our proposed conversion between gene copy number and cercariae number utilizes the conversion equation and accounts for losses due to DNA extraction. We opted to use a standard curve to predict the number of cercariae rather than using a single number to estimate, as interpolating based on a standard curve better accounts for uncertainty and variability in the target measurement compared to using a single-point measurement. Extraction efficiencies between 0.5 and 4% have been reported for bacteria (Hwang et al. 2012). Therefore, it is not surprising that DNA extraction efficiencies ranged between 1 and 7% (Table 3). These results demonstrate how imperative it is to quantify losses through the extraction procedure in order to avoid underestimations in target concentrations. Other studies that have used microscopic cercariometry to quantify cercariae abundance have found as many as between 200 and 300 cercariae/L in still-water environments (Theron et al. 1978), while typical values in a more dynamic system range typically average 10 cercariae/L (Aoki et al. 2003).

Accuracy of the qPCR methods was tested in a single blinded study. Using the conversion equation and an estimation extraction efficiency of 4.2%, the actual number of cercariae was estimated correctly in each vial, suggesting that under ideal circumstances (i.e., purified cercariae in a clean matrix), this method of estimating cercariae concentrations from qPCR copy numbers is accurate and robust (Fig. 1B). Precision was tested in the field by collecting 3 consecutive 25-L water samples at ten beaches from Crystal Lake in 2016. Variation between samples in this dynamic natural environment is low, with the highest samples exhibiting variation of between 8 and 12 cercariae in between replicate samples. These results demonstrate that the assay provides a precise estimate of cercariae concentration over time in a natural environment (Fig. 1C), but that multiple samples will result in more robust estimation of cercariae concentrations at a particular location.

### Environmental and Seasonal Drivers of Cercariae Abundance

Laboratory studies have demonstrated that cercariae of *Trichobilharzia* are positively phototactic and negatively geotactic, and the parasites are typically shed in the morning (Feiler and Haas 1988; Haas et al. 2008). Given these biological determinants of cercariae behavior, it could be expected that avian schistosome cercariae will be more likely to be found during the morning and in the topmost column of the water (Feiler and Haas 1988). Results of the water depth study demonstrated that the majority of cercariae reside in the top 15–20 cm of the water (Fig. 1D), which confirms observations made by others in the laboratory (Feiler and Haas 1988). The time of day experiment shows that on average, more cercariae are found in the water earlier in the day, between 8 a.m. and 12 p.m., and gradually decline throughout the day (Fig. 1E). However, the majority of samples collected on this day were collected during onshore or alongshore winds; therefore, it remained unclear if wind, or the time of sample collection may be responsible for the higher concentrations of cercariae observed between 8 a.m. and 12 p.m.

To better elucidate the biological and environmental drivers of cercariae abundance, a generalized linear model was performed on the entire dataset from 2016 to 2017. Results of the generalized linear model demonstrated the importance of onshore, and alongshore wind in explaining higher cercariae concentrations. Time of sampling (morning or afternoon) did not explain higher cercariae concentrations. There were 2 weeks, corresponding to early July and early August, which also predicted higher numbers of cercariae that could be due to seasonal trends in patent infections among snails (Table 4). Wind conditions likely blow cercariae closer to shore, as such it is intuitive that on- and alongshore winds would increase concentrations of cercariae along the shore. This is intuitive given that our study has also confirmed laboratory experiments that cercariae position themselves in the topmost portion of the water body, and therefore, their movement in the water is likely influenced greatly by surface winds, akin to the movement of surface cyanobacteria scums (Kahru et al. 2007; Kanoshina et al. 2003). Wind direction has also been previously implicated in moving *Trichobilharzia* cercariae (Leighton et al. 2000). Our model suggests that time of day does not have a strong relationship with cercariae abundance. However, as avian trematodes do leave their snail hosts in the morning, early morning bathers may still be at greater risk of encountering high concentrations of cercariae. There are numerous reports in the literation of strong seasonal trends in snails shedding cercariae, and this likely explains the higher overall cercariae concentrations found in weeks 4 and 8. Numerous studies have reported seasonal trends in patent infections in snails (Crews and Esch 1986; Brown et al. 1988; Gordy et al. 2016). It must be acknowledged that we tested a few factors which might affect cercariae concentrations—however, there are a number of potentially important variables which might influence cercariae concentrations, including water temperature, unpredictable cercariae shedding from snails, and shoreline bird abundance (Lo and Lee 1996; Abrous et al. 1999; Byers et al. 2008; Soldánová et al. 2016). Furthermore, qPCR as a DNA-based molecular method will detect both live and dead cercariae in the water column and therefore may overestimate infection risk in some instances (Bass et al. 2015).

The continued development of molecular testing for environmental stages of parasite development is essential to fully elucidating and understanding transmission and infection risks in parasitic lifecycles (Bass et al. 2015). We hope that our contributions to the avian schistosomes qPCR cercariometry method enable others to continue to elucidate environmental and biological drivers of infection in a variety of host parasite systems. Here we confirm a few previously hypothesized drivers of infection risk, but with this method, a variety of other hypotheses can be tested in the future.

This study demonstrates the utility of these molecular methods more broadly. For instance, analogous methods could be applied to tracking the spatial and temporal changes in *Schistosoma mansoni*, or *Schistosoma japonicum* cercariae which cause human schistosomiasis. While qPCR tests have been developed for both of these species, and have been trialed in ambient waters, to our knowledge no comprehensive assessment of cercariae concentrations and conditions that may increase or decrease their concentrations has been undertaken (Hertel et al. 2004; Hung and Remais 2008). Such an assessment is a natural next step for both DNA-based parasitological testing, but also could aid in monitoring the effectiveness of control efforts (Melo et al. 2006).

## Electronic supplementary material

Below is the link to the electronic supplementary material.

Supplementary Figure 1 A diagram of the stacked V trap water column-sampling device used to sample the number of parasites at different depths in the water column. (TIFF 850 kb)
