# Supplementary figures and images for: Use of qPCR-Based Cercariometry to Assess Swimmer’s Itch in Recreational Lakes
Source: Ecohealth. 2018 Aug 17;15(4):827–39. doi: 10.1007/s10393-018-1362-1 (PMC6267424; doi:10.1007/s10393-018-1362-1)

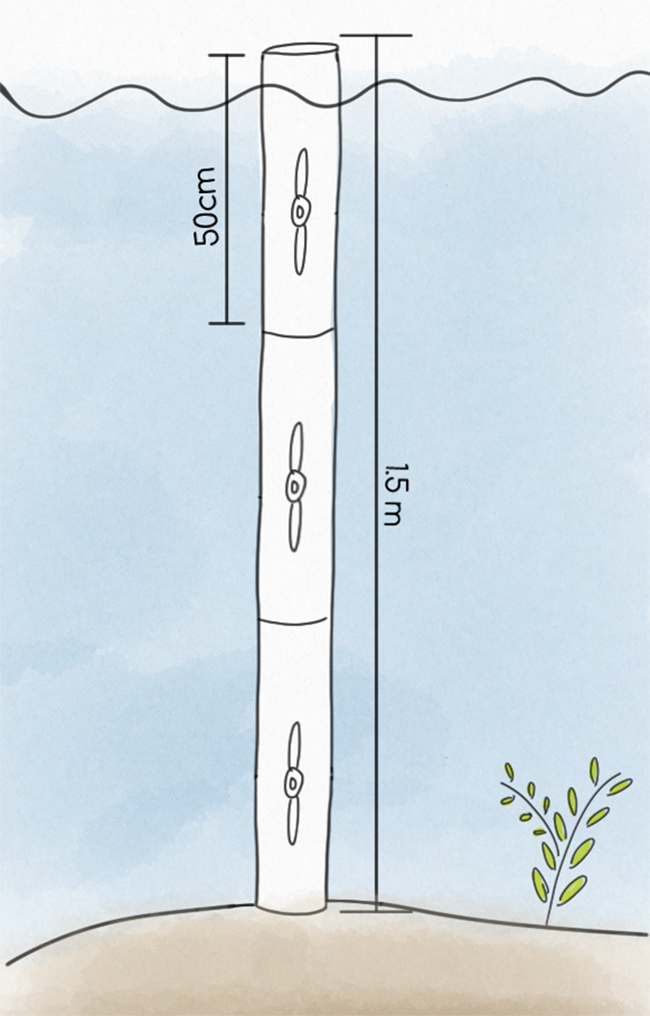

Supplement: Supplementary file 1 — A diagram of the stacked V trap water column-sampling device used to sample the number of parasites at different depths in the water column. (TIFF 850 kb) [file 10393_2018_1362_MOESM1_ESM.tif]
